# One Health Approach to *Sporothrix brasiliensis*: Disinfection Strategies for Control and Prevention

**DOI:** 10.1590/0037-8682-0284-2025

**Published:** 2026-02-09

**Authors:** Regielly Caroline Raimundo Cognialli, Flávio Queiroz-Telles, Giovanna Carnieri Daitschman Santos, Felipe Moreira Matias, Luciano Moreira, Elias Nunes Monteiro, Mônica Surek, Vânia Aparecida Vicente, Izabella Castilhos Ribeiro dos Santos-Weiss

**Affiliations:** 1 Universidade Federal do Paraná, Hospital de Clínicas, Curitiba, PR, Brasil.; 2 Universidade Federal do Paraná, Departamento de Patologia Básica, Programa de Pós-Graduação em Microbiologia, Parasitologia e Patologia, Ciências Biológicas, Curitiba, PR, Brasil.; 3 Universidade Federal do Paraná, Departamento de Saúde Pública, Curitiba, PR, Brasil.; 4 Universidade Federal do Paraná, Curso de Farmácia, Curitiba, PR, Brasil.; 5 Universidade Federal do Paraná, Departamento de Análises Clínicas, Curitiba, PR, Brasil.; 6 Universidade Federal do Paraná, Departamento de Patologia Básica, Curitiba, PR, Brasil.

**Keywords:** Decontamination, Sporotrichosis, Transmission, Environmental, Disinfectant agents

## Abstract

**Background:**

Sporotrichosis is a neglected tropical disease caused by several species of *Sporothrix*, of which *S. brasiliensis* is the most virulent. Transmission primarily occurs through scratches, bites, or direct contact with exudates from infected cats and potentially through fomites.

**Methods:**

We evaluated the efficacy of different disinfectant solutions in eliminating *S. brasiliensis* from contaminated surfaces, including medium-density fiberboard (MDF), tiles, stainless steel, and polyester fabric, by simulating both domestic and clinical environments. Sterile 9-cm² sections were uniformly inoculated with a standardized yeast-phase suspension. After drying, the surfaces were treated with 70% ethanol, sodium hypochlorite, quaternary ammonium compounds, or laundry soap (fabric only). Samples were collected at 0-30 min and cultured in triplicate on Mycosel medium at 30°C. Fungal viability was determined microscopically to assess the disinfection efficacy.

**Results:**

Based on our results, 70% ethanol and bleach were the most effective disinfectants and recommended for use in both household and clinical settings. Bleaching displayed the highest efficacy, whereas 70% ethanol performed well. Other disinfectants demonstrated variable effectiveness depending on the exposure time and surface type.

**Conclusion:**

This study highlights the critical role of disinfection in mitigating the environmental transmission of sporotrichosis, thereby safeguarding both human and animal health.

## INTRODUCTION

Sporotrichosis is a neglected tropical disease caused by different *Sporothrix* species^1^ ^,^ ^2^. Among these, *Sporothrix brasiliensis* is the most virulent. Although transmission primarily occurs through scratches or bites from infected cats, sapronotic- and fomite-mediated transmission has also been documented^3^ ^-^ ^5^. *S. brasiliensis* has emerged as the primary etiological agent of expanding zoonotic mycosis in South America. It was initially identified in Brazil, with subsequent cases reported in other countries^6^ ^-^ ^15^. 

Zoonotic transmission by infected cats typically occurs through traumatic inoculation via scratches and bites; however, it can also be transmitted through direct contact with exudates and respiratory droplets^16^ ^-^ ^18^. Recently, it has been reported that fomites may represent a potential route for *S. brasiliensis* transmission^5^ ^,^ ^19^. Infected cats present with a high fungal burden on their lesions^20^ ^,^ ^21^, which can contaminate the environment and surfaces^19^ ^,^ ^22^, thereby infecting other cats, humans, and dogs. It is essential to identify effective disinfectants to minimize environmental contamination and the potential for fomite transmission. 

Sporotrichosis, caused by *S. brasiliensis* is a significant public health concern affecting thousands of humans and animals^6^ ^,^ ^7^ ^,^ ^23^ ^,^ ^24^. Domestic cats play a key role in disease epidemiology because of their high fungal burden and close contact with humans^20^ ^,^ ^21^. In this context, it is crucial to improve the diagnostic and treatment methods for these animals while strengthening prevention efforts. These measures should include the use of personal protective equipment when handling infected animals, the cremation of infected animal carcasses, and proper disinfection of materials and environments^25^. 

A One Health approach is essential to prevent and control sporotrichosis, requiring collaboration across the human, animal, and environmental health sectors^17^. However, significant challenges remain, particularly in the effective disinfection of surfaces contaminated with *S. brasiliensis*. This is particularly important to prevent transmission through fomites, particularly in veterinary clinics and households with infected cats^19^. By reducing the likelihood of indirect exposure in both humans and animals, appropriate disinfection practices contribute to broader prevention efforts and reinforce the One Health perspective, which integrates environmental, animal, and human health. This study evaluated the efficacy of common disinfectants on surfaces contaminated with *Sporothrix brasiliensis* to support evidence-based protocols for preventing environmental and zoonotic transmission.

## METHODS

A prospective experimental study was conducted to evaluate the efficacy of different cleaning solutions for chemical disinfection of inanimate surfaces contaminated with *S. brasiliensis*. This study used a new protocol developed by the authors to evaluate disinfectants against *S. brasiliensis* using a simple methodology that simulates real-world conditions. Different types of surfaces were selected to simulate common environments, including medium-density fiberboard (MDF) wood, tiles, stainless steel, and polyester fabric. MDF wood and tiles were selected to mimic household environments, whereas stainless steel and polyester fabrics were selected to mimic clinical and laboratory environments. All surfaces were divided into standardized and clearly delimited quadrants of approximately 9 cm². For the polyester fabric, the quadrants were cut and individually separated to ensure consistent delimitation across samples. The tile, wood, and fabric materials were sterilized using ultraviolet (UV) light, whereas the stainless-steel surface was sterilized by autoclaving. The isolate CMRP6191 of *S. brasiliensis* characterized by molecular biology, was selected for this study from the Microbiological Collections of the Paraná Network-Taxonline (CMRP/Taxonline-https://www.cmrp-taxonline.com/). Sequence data are available in GenBank under the accession number PV740346.

The disinfectant solutions selected included products commonly used in healthcare facilities in Brazil, as well as those accessible to the general public^26^. These were 70% ethanol, bleach (Qboa®; active ingredient: sodium hypochlorite), 0.5% Surfic® (solution X-active ingredients: 5.2% polyhexamethylene biguanide and 0.5% benzalkonium chloride), Lysoform® (solution Y-active ingredient: 0.45% n-alkyl dimethyl benzyl ammonium chlorides), and laundry soap (Tixan Ipê®), the last one tested only on polyester fabric. Although bleach and Solution Y are commercially available to the general public, 70% ethanol and Solution X are restricted to healthcare facilities in Brazil.

*Sporothrix* species are thermodimorphic fungi, exhibiting the yeast phase at 35-37°C and the mycelial phase at 25-30°C. Because surface contamination is associated with the yeast phase present in lesions of infected cats, *S. brasiliensis* was cultured in Brain Heart Infusion (BHI) medium and incubated at 37°C for 7 days to revert to the yeast phase. Subculture was performed in the BHI medium under the same conditions for an additional 7 days to fully revert to the yeast phase. A suspension of the yeast phase of *S. brasiliensis* was prepared in sterile saline to a final volume of 600 mL with a turbidity of 1.42. Cell counting using a Neubauer chamber was not performed; however, based on a previous study that used *S. brasiliensis* in the yeast phase^27^, the concentration can be estimated to range from 2.8 × 10⁶ to 1.4 × 10⁷ CFU/mL. Before inoculation with the fungal suspension, all surfaces were examined for microbial growth in triplicate, and no fungal colonies were detected. This procedure served as a negative control to confirm that the surfaces were sterile before contamination, ensuring that any subsequent growth resulted solely from the experimental inoculum. All surfaces were then uniformly contaminated with the suspension using a Pasteur pipette, applying a volume corresponding to the amount necessary to fully cover each surface quadrant (approximately 1 mL per quadrant), to ensure uniform spreading of the fungal suspension across all surface types. The surfaces were allowed to dry and were maintained at room temperature and humidity.

**FIGURE 1:**
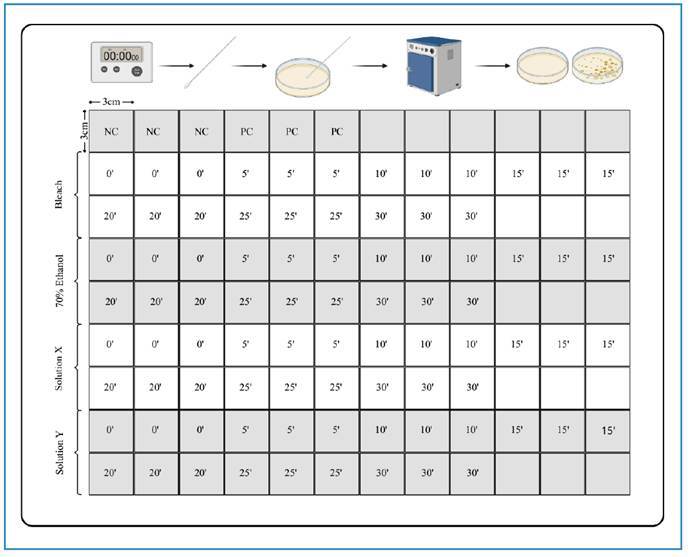
Schematic representation of the sample collection procedure. **Legend:** This figure illustrates the methodology used in this study. Each quadrant measured 9 cm². The first row shows controls: NC, negative control; PC, positive control. The following rows show the quadrants designated for each disinfectant tested (bleach, 70% ethanol, solution X, and solution Y). Analyses were performed in triplicate at each time point (0, 5, 10, 15, 20, 25, and 30 min). This procedure was repeated for all surfaces tested, except for the polyester fabric, as mentioned above.

For the disinfection test, the MDF wood, tiles, and stainless steel were cleaned with a sterile gauze using each of the tested cleaning solutions until the entire surface was in contact with the product. The bleach was diluted to final concentrations of 0.1% sodium hypochlorite for the surface and 0.02% for the polyester fabric. The laundry soap was diluted to a concentration of 15 g/L. The polyester fabric was dipped into a container containing a solution of 0.02% NaOCl and laundry soap. Fungal cultures were conducted in triplicate on Mycosel medium (BBL) both before and after cleaning the surfaces with the products. Samples were collected using sterile cotton swabs from different quadrants of each material at each predefined time point. Sampling was performed in triplicate at 0, 5, 10, 15, 20, 25, and 30 min, with the 0-min time point corresponding to the samples taken immediately after cleaning (Figure 1). Cultures were incubated at 30ºC, monitored daily, and fungal growth was assessed through microscopic analysis.

## RESULTS

*Sporothrix* growth was observed in all fungal cultures used as positive controls for contamination. We observed consistent results across all triplicates and different outcomes depending on the disinfectant agent, exposure time, and surfaces evaluated.

As shown in Table 1, the results demonstrated that when 70% ethanol was used to disinfect the stainless-steel and MDF wood surfaces, no growth of *S. brasiliensis* was observed after 5 min of exposure. In contrast, on tile surfaces, fungal growth persisted throughout the entire 30-min evaluation period. Disinfection with bleach was effective after 5 min on stainless steel, tile, and MDF wood surfaces. However, the same performance was not observed for polyester fabric, for which complete disinfection was achieved after only 30 min. For solution X, disinfection occurred after 15 min on the stainless steel and MDF wood surfaces, whereas the tile surfaces required 25 min. Solution Y effectively disinfected stainless steel, tile, and MDF wood surfaces after 15 min of exposure. Laundry soap demonstrated no disinfectant activity on polyester fabric within the 30-min period evaluated in this study.

**TABLE 1:** Results of the disinfection experiment against *S. brasiliensis* on different surfaces using 70% ethanol, bleach, solution X, solution Y and laundry soap.

		Stainless steel	Tiles	Medium-density fiberboard wood	Polyester fabric
**Disinfection solution**	Time				
**70% Ethanol**	T0’	+	+	+	NE
	T5’	-	+	-	NE
	T10’	-	+	-	NE
	T15’	-	+	-	NE
	T20’	-	+	-	NE
	T25’	-	+	-	NE
	T30’	-	+	-	NE
Bleach*	T0’	+	+	-	+
	T5’	-	-	-	+
	T10’	-	-	-	+
	T15’	-	-	-	+
	T20’	-	-	-	+
	T25’	-	-	-	+
	T30’	-	-	-	-
**Solution X**	T0’	+	+	+	NE
	T5’	+	+	+	NE
	T10’	+	+	-	NE
	T15’	-	+	-	NE
	T20’	-	+	-	NE
	T25’	-	-	-	NE
	T30’	-	-	-	NE
**Solution Y**	T0’	+	-	+	NE
	T5’	+	-	+	NE
	T10’	-	-	+	NE
	T15’	-	-	-	NE
	T20’	-	-	-	NE
	T25’	-	-	-	NE
	T30’	-	-	-	NE
**Laundry soap**	T0’	NE	NE	NE	+
	T5’	NE	NE	NE	+
	T10’	NE	NE	NE	+
	T15’	NE	NE	NE	+
	T20’	NE	NE	NE	+
	T25’	NE	NE	NE	+
	T30’	NE	NE	NE	+

**Legend:** (*) 0.1% NaOCl for stainless steel, medium-density fiberboard wood, and tiles, and 0.02% NaOCl for polyester fabric. The symbols (+) and (−) stand for growth and no growth in fungal culture, respectively. **NE:** not evaluated.

**FIGURE 2:**
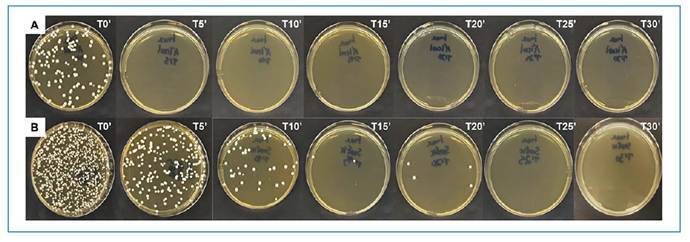
Disinfection test on stainless steel with 70% ethanol and solution X. **Legend:** Fungal cultures showing the growth of *S. brasiliensis*. **Line A:** Disinfection test using 70% ethanol on stainless steel at 0, 5, 10, 15, 20, 25, and 30 min after disinfection. **Line B:** Disinfection test using solution X on stainless steel at 0, 5, 10, 15, 20, 25, and 30 min after disinfection.

Bleaching yielded the best results across all surfaces analyzed. Solution Y provided satisfactory outcomes with a minimum contact time of 15 min. Good performance was observed with 70% ethanol on stainless steel and MDF wood; however, it was less effective on tiles. Among all disinfectants evaluated, solution X displayed the poorest performance. For the polyester fabric, which simulated laboratory coats, disinfection was achieved after only 30 min of soaking in 0.02% bleach. The results of the efficacy tests for 70% ethanol and Solution X on the stainless steel are shown in Figure 2 **.**

## DISCUSSION

Zoonotic sporotrichosis affects individuals of all ages and socioeconomic backgrounds, although it disproportionately impacts lower-income populations^6^ ^,^ ^28^ ^,^ ^29^. Consequently, selecting disinfectants requires a balance of efficacy, safety, cost-effectiveness, and local availability to ensure equitable adoption in diverse socioeconomic contexts. Disinfection can be achieved through physical or chemical methods, each offering distinct advantages depending on the context and surface to be treated^30^. 

Our findings are consistent with the recommendations described in the Technical Note from the Ministry of Health of Brazil (technical note no. 60/2023-CGZV/DEDT/SVSA/MS), which advises cleaning environments and equipment used for animal handling, such as transport boxes, with disinfectants containing quaternary ammonium compounds in the dilutions recommended by the manufacturer, 70% ethanol, and 1% sodium hypochlorite. In addition, the technical note recommends washing blankets with water and soap, and disinfecting food and water bowls, as well as any other items that have direct contact with infected animals^31^. These recommendations, based on the practical experience in handling pathogenic agents, are supported by the experimental evidence generated in this study. However, the lack of standardized efficacy studies for some of the recommended disinfectants, particularly in the context of *Sporothrix* spp., highlights the need for further experimental validation and underscores the relevance and importance of the present study.

Ethanol-based products are commonly used as disinfectants for microorganisms owing to their low toxicity^32^. In Brazil, 70% ethanol is not commercially available to the public and is restricted to healthcare facilities. Technical Note number 60/2023 recommends the use of 70% alcohol for the disinfection of equipment used in animal handling^31^. Based on our results, 70% ethanol is effective against stainless steel and MDF wood after 5 min. One study demonstrated that *S. brasiliensis* can persist for up to 10 days on MDF and 25 days on stainless steel^5^. More recently, another study reported the isolation of *Sporothrix* from stainless-steel surfaces, including procedure tables and cages, in veterinary facilities specializing in treating cats with sporotrichosis^19^. Therefore, it could be an excellent choice for veterinary clinics, microbiology laboratories, and public institutions involved in handling infected animals, such as zoonotic surveillance and animal welfare organizations. Surface characteristics such as porosity and surface texture can impair disinfectant contact and penetration, which possibly explains the reduced efficacy of 70% ethanol observed on tiles. Previous studies reported lower log reductions on porous or textured materials and recommended longer contact times, mechanical removal, or alternative agents/coatings for effective decontamination^33^.

Bleach (sodium hypochlorite) is a chlorine-based compound that is widely used in domestic environments^32^ ^,^ ^34^. Our results demonstrated that bleach was the most effective disinfectant solution for all tested surfaces. For the polyester fabric, bleach was the only solution capable of achieving disinfection, although it had the drawback of discoloring the fabric. Given this limitation, the use of a disposable lab coat could be considered an alternative to reduce the risk of contamination. The *in vitro* susceptibility of *Sporothrix* to 4% sodium hypochlorite has been previously evaluated using disc diffusion, broth microdilution, and direct exposure tests, all of which have demonstrated its high antifungal potential^35^. For zoonotic sporotrichosis, surface disinfection was performed with 1% sodium hypochlorite for 10 min^25^ ^,^ ^31^. However, the toxicity of NaOCl should be considered depending on the concentration, exposure route, and duration, especially for inhalation and dermal exposure^34^. Nonetheless, these effects were minimized because of the low concentration of sodium hypochlorite tested (0.1% and 0.02%), with no or minimal/transient effects, even in humans and animals^34^ ^,^ ^36^. Our findings support the use of diluted bleach (0.1-0.02%) as a cost-effective and safe solution for households in accordance with Brazil’s public health guidelines. Our data support that these lower concentrations (0.1-0.02%) match the disinfection efficacy of the 1% reference^31^, offering practical, environmental, and operational advantages. Low-concentration bleach solutions are far less toxic to humans, animals, and ecosystems, yet remain microbicidal at levels shown to be effective in other studies, reinforcing our One Health-aligned approach and advocating evidence-based updates to disinfection protocols.

Solution X is a quaternary ammonium compound (QAC) containing benzalkonium chloride, which is commonly used as a potent disinfectant, particularly in hospitals. Solution X demonstrated limited effectiveness on the tested surfaces and required prolonged exposure times. Solution Y, which is also a QAC, is commercially available for domestic use and demonstrates excellent performance, particularly in tiles. Concerns exist regarding bacterial tolerance or resistance to QACs, as well as their toxicity and potentially harmful effects on humans and the environment, especially on aquatic organisms^36^ ^-^ ^38^, which underscores the need for exercising care in their use and disposal. The risks of exposure to or ingestion of QACs are generally considered low in veterinary medicine; however, fatal exposures in dogs^39^. In addition, solution X contained benzalkonium chloride, which is associated with toxicity in cats and causes chemical burns and oral/esophageal ulceration^36^. Given these concerns, the use of this solution for *S. brasiliensis* disinfection should be carefully evaluated.

The laundry soap commonly used in households was not effective on the fabric within the contact time assessed in this study. Given its frequent use in domestic settings, it is important to emphasize that laundry soap alone, with only 30 min of contact with the fabric, is not sufficient for effective disinfection. In this context, for fabric, we suggest evaluating its performance with longer contact times and considering its use in combination with the other agents assessed in this study that demonstrated greater disinfection efficacy.

It is essential to emphasize several key points: all cleaning products should be applied with appropriate levels of caution, and appropriate personal protective equipment, such as gloves and masks, should be used. Cats are deficient in the enzyme UDP-glucuronosyltransferase^40^, making them highly susceptible to the toxicity of phenol-based disinfectants and essential oils^36^. Therefore, these products were not tested and should be avoided. To protect animals and prevent toxicity from disinfecting agents, individuals responsible for cleaning contaminated areas should avoid performing cleaning procedures in the presence of infected animals. The animal was temporarily relocated to a separate area until the cleaning was complete.

Other disinfection practices may also be useful; however, they were not evaluated in this study. For example, ultraviolet light C (UVC) was tested against *S. brasiliensis* in both its yeast and filamentous phases, demonstrating excellent performance, with a reduction of 78 to 100% in the fungal burden^30^. Despite these promising results, UVC disinfection remains restricted to specialized groups, such as researchers and healthcare professionals, with access to this technology, and is not yet applicable to the general population.

A key limitation of this study is the use of a single *S. brasiliensis* isolate, which may not fully represent the genetic diversity of the species. Nevertheless, the experimental protocols were carefully designed to mimic the real-world conditions in Latin American countries, thereby increasing the translational relevance of the findings. These results provide valuable insights with important public health implications, particularly for the prevention and management of sporotrichosis in Brazil.

Knowing which disinfectants are effective against *S. brasiliensis* on surfaces is crucial not only for the general public but also for veterinary professionals, physicians, pharmacists, microbiologists, and other healthcare workers. This study helps fill an important gap in our understanding of how to prevent the environmental transmission of *S. brasiliensis* through fomites, thereby protecting both humans and animals from potential infections.

## Data Availability

Data-in-article: Research data is available in the body of the document (tables and figures).
